# Recent Advances in the Photonic Curing of the Hole Transport Layer, the Electron Transport Layer, and the Perovskite Layers to Improve the Performance of Perovskite Solar Cells

**DOI:** 10.3390/nano14100886

**Published:** 2024-05-19

**Authors:** Moulay Ahmed Slimani, Sylvain G. Cloutier, Ricardo Izquierdo

**Affiliations:** Département de Génie Électrique, École de Technologie Supérieure, 1100 Rue Notre-Dame Ouest, Montréal, QC H3C 1K3, Canada; moulay-ahmed.slimani.1@ens.etsmtl.ca (M.A.S.); sylvaing.cloutier@etsmtl.ca (S.G.C.)

**Keywords:** photonic curing, ETL, HTL, perovskite solar cell

## Abstract

Perovskite solar cells (PSCs) have attracted increasing research interest, but their performance depends on both the choice of materials and the process used. The materials can typically be treated in solution, which makes them well suited for roll-to-roll processing methods, but their deposition under ambient conditions requires overcoming some challenges to improve stability and efficiency. In this review, we highlight the latest advancements in photonic curing (PC) for perovskite materials, as well as for hole transport layer (HTL) and electron transport layer (ETL) materials. We present how PC parameters can be used to control the optical, electrical, morphological, and structural properties of perovskite HTL and ETL layers. Emphasizing the significance of these advancements for perovskite solar cells could further highlight the importance of this research and underline its essential role in creating more efficient and sustainable solar technology.

## 1. Introduction

Photonic curing (PC) is a method that is employed in several sectors, most notably printed electronics and coatings. It is a process of curing or drying inks, coatings, and other materials that employs powerful light pulses. Curing light is often in the ultraviolet (UV) and infrared (IR) spectra, as well as flash white light [1]. PC is a specialized process for producing nanoparticle-based thin films. The procedure involves using intense laser or light irradiation to rapidly cure and heat the material. It consists of three steps: (1) preparing the ink or paste, (2) drying the ink through a solvent elimination process, and (3) exposing the material to powerful light to generate a dense film.

Since 2006, NovaCentrix has led to advancements in printed electronics, including the introduction of the first commercial PC device. This technology has since evolved through innovations in flashlamp technology, digital control, and pulse tuning [2]. The PC rapidly raises the temperature of the material and causes the solvent to evaporate, eventually producing a solid, cured coating. PC is widely used in the development of various types of printed electronics, including flexible displays, thin-film transistors [3], RFID tags [4,5], sensors, and solar cells [6,7], but it is also increasingly used in the industrial sector, in the automotive, and solar industries [8,9] to enhance production and improve performance. PC is especially useful for printing on heat-sensitive substrates such as plastic or paper because it reduces heat exposure and potential substrate damage; the material’s temperature rises rapidly as an outcome of this exposure. The high-intensity light generates enough energy to start and finish the curing process in milliseconds, making it substantially faster than traditional thermal annealing (TA). PC is being researched and developed all the time, and it has the potential to change manufacturing processes in a variety of industries, making them more efficient and ecologically friendly. PC has evolved into a specialized technique for treating surfaces, presenting its importance in perovskite solar cell manufacturing through advancements like optimizing curing parameters, adapting to delicate substrates, controlling layer morphology, and integration with manufacturing processes. Recently applied in perovskite solar cell fabrication, PC’s potential for high efficiency and low processing cost is explored in this review, focusing on enhancing HTL, ETL, and perovskite material crystallization to improve the fabrication and processing of perovskite solar cells (PSCs).

## 2. Photonic Curing of the HTL Layer

Photonic annealing of HTL layers is an innovative and promising substitute for TA. It reduces the requirement for complex and time-consuming processes, making it a precise and efficient approach that has recently garnered significant interest. It is an annealing process using intense pulsed light (IPL). A brief pulse (25–100 ms) of broad-spectrum light (0.2–1.5 μm) turns energy into heat inside light-absorbing layers [10]. The pulse intensity could be high; however, the cumulative energy is low due to the brief exposure [9]. To our knowledge, there has not been enough study of the photonic annealing of HTL, excluding sol-gel precursors, which were only used by the Hsu group as HTLs for flexible and rigid perovskite solar cells [8,9]. Similar to perovskite and other inks (${\mathrm{TiO}}_{2}$, ${\mathrm{SnO}}_{2}$, ${\mathrm{ZrO}}_{2}$), converting sol-gel precursors into a NiOx oxide film on a flexible substrate, presents many challenges. Nickel nitrate precursors are transparent, requiring higher energies, which could damage the flexible film; moreover, previous work has shown that NiOx is not a crystalline structure and remains transparent after photonic annealing, making it difficult to determine the degree of conversion of NiOx. To address this challenge, the ITO-coated flexible Corning Willow Glass (WG) substrate is used for the annealing process [11]. This option allows for roll-to-roll manufacturing compatibility and facilitates the comparison of both thermal and PC methods. In addition, low-cost solution methods (solution-based NiOx nanoparticles [12]) can also be employed to prepare efficient NiOx hole contacts. However, their application in flexible solar cells is restricted by the annealing process required at 300–500 °C [13,14]. In addition, developing a one-step process to convert NiOx from its sol-gel precursor is an interesting approach. Hsu’s group has proven the possibility of creating a dense semiconducting metal oxide film from a sol-gel precursor through PC [9]. The choice of sol-gel nitrate precursors is justified by their lower decomposition temperature compared to other NiOx precursors, thus requiring less energy to convert the precursors into a dense film [15,16]. In their study, the authors compared the efficiency of various configurations of perovskite solar cells (PSCs) using thermal and photonic annealing for both NiOx HTL and perovskite active layer. All PC MAPI samples were treated with a single pulse at a lamp voltage of 300 V and a pulse duration of 20 ms, but the PC NiOx Champion was processed using a lamp voltage of 500 V for 10 ms with 10 pulses at a frequency of 0.2 Hz [9].

**Figure 1:**
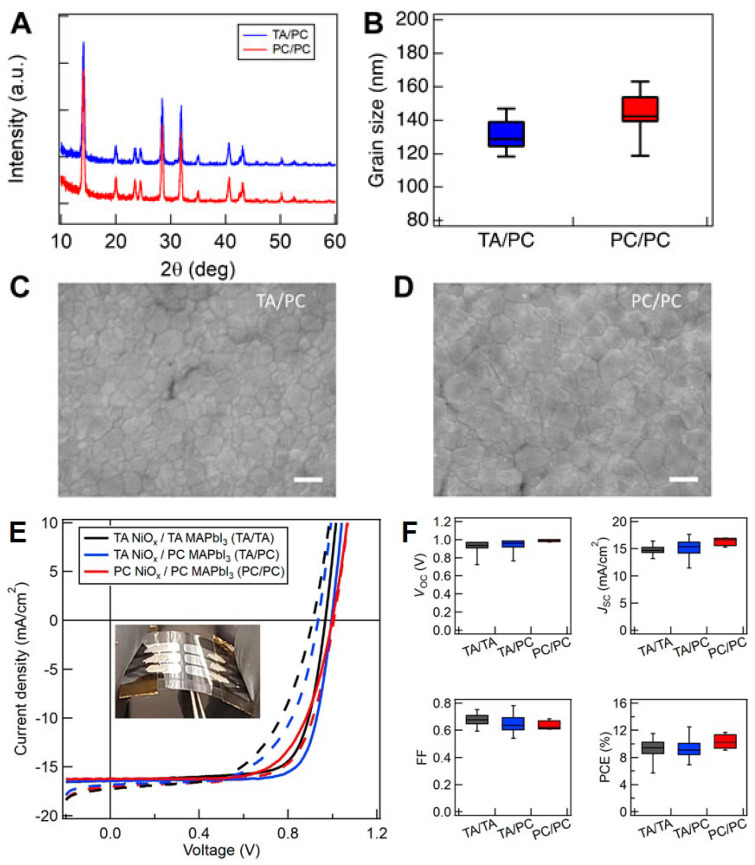
(**A**) XRD of PC MAPI samples on TA and PC NiOx. (**B**) Grain size analysis of MAPI SEM images. SEM images of PC MAPI films on (**C**) TA NiOx and (**D**) PC NiOx, respectively. (**E**) J-V measurements of flexible PSCs Reverse Champion (solid) and forward scan (dashed). (**F**) Box plots of V_oc_, J_sc_, FF and PCE variations for PSCs fabricated under ambient conditions (from [9]).

XRD analysis (Figure 1) showed that the crystal phase of perovskite was comparable for TA and PC, as were the microstructure, grain size, and topography (Figure 1B–D). TA and PC are applied to both NiOx and MAPI, enabling three different solar cell configurations: (1) TA NiOx and TA MAPI (TA/TA), (2) TA NiOx and PC MAPI (TA/PC), and (3) PC NiOx and PC MAPI (PC/PC). The maximum power conversion efficiencies (PCEs) are achieved by the TA/PC configuration (see Table 1). However, for the average PCE, we can see a slight improvement with the PC/PC and low dispersion compared to the other configurations. This is probably explained by the fact that PC, as opposed to TA, can provide excellent processing uniformity because the light source can be controlled very precisely, allowing the rapid and uniform heating of the material surface.

Table 1 presents a comparison of the maximum PCE of PSCs using NiOx. As shown in Table 1, PC treatment gives a similar maximum power conversion efficiency to conventional TA, which is consistent with previous studies [17]. Similarly, the PSC performance results were similar for TA and PC. (Figure 1E,F). In addition, PC testing has shown that a single pulse can convert NiOx and MAPI films into dense, uniform films with excellent electrical properties and is sufficient to achieve maximum performance for both NiOx and perovskite. TA is conducted in a glove box, while PC is performed under ambient conditions; however, their performance is comparable.

The Hsu group’s research focuses on the use of NiOx as an HTL layer for PSC on flexible and glass substrates [8,9]. This work examines the impact of photonic curing on PSC efficiency, demonstrating how it can reduce production time and costs by rapidly heating and crystallizing the material. In addition, the group is examining the potential of photonic curing in perovskite solar cells, particularly in the context of high-throughput manufacturing.

## 3. Photonic Curing of ETL Layer

Moreover, beyond HTL, photonic curing has been employed on various materials utilized in the production of PSCs, such as ETL. The preparation of the different HTL layers, the active layer, and the ETL layer requires sequence deposition and annealing, which sometimes limits the use of certain materials (ETL, HTL) and substrates due to high temperatures. ${\mathrm{TiO}}_{2}$ is typically used in mesoporous [18,19] or planar structures in PSCs [20,21]. It is widely used in standard configurations as an ETL layer in PSCs. However, its crystallization into a compact layer requires a high temperature (400–500 °C) [22,23], which is not compatible with certain PSC configurations (p-i-n) or with flexible plastic-based substrates (kapton, polyethylene terephthalate (PET), poly-ethylene naphthalate (PEN)). To overcome these issues, various approaches have been developed for fabricating compact ${\mathrm{TiO}}_{2}$ coatings for PSCs, such as sputtering process [24,25], which showed power conversion efficiencies (PCEs) of 16.1% and 23% on glass substrates; electron beam evaporation [26], demonstrating 11.6%; and solution treatment methods, involving a lower temperature of 150 °C, which resulted in a PCE of 13.5% [27]. In contrast to these techniques, the PC of ${\mathrm{TiO}}_{2}$ is promising mainly because of its compatibility with R2R and sensitive substrates (PEN, PET) [28], Additionally, its reduced process time and low thermal budgets [29] make it appealing for large-scale industry. Several studies have confirmed that the use of PC is an effective technique to achieve the improved crystallization of ${\mathrm{TiO}}_{2}$ films on glass and plastic substrates that are coated with ITO or FTO. The extremely short pulse duration emitted by the IPL system is sufficient to sinter the ${\mathrm{TiO}}_{2}$ without damaging the substrate. It has been shown that surface pretreatment is necessary to replace high temperatures for ${\mathrm{TiO}}_{2}$ crystallization by reducing surface tension, facilitating wetting, and decomposing the nitrate resulting from synthesis [30]. IPL, on the other hand, requires no additional treatment to eliminate the nitrate binder, which negatively affects the efficiency of PSCs [28]. In the same study, Table 1 illustrates that both IPL and TA exhibit similar performance for all configurations within the energy density range of 10 to 20 mA cm^−2^ and pulse duration range of 2 to 7 ms for glass substrates. However, the energy density needed for PEN substrates is less (2.35 mA cm^−2^) than that of glass substrates due to their higher absorbance capacity. On flexible PEN substrates, the average yield reaches 10%, which is an outstanding achievement for this type of configuration and appealing for R2R applications.

**Figure 2:**
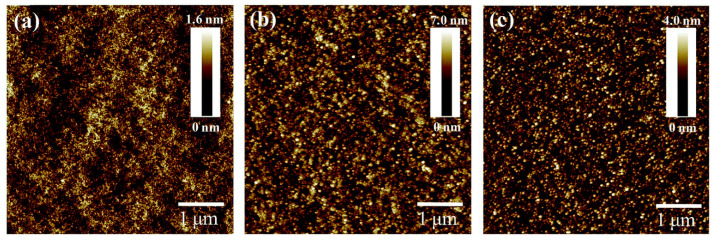
AFM images for ${\mathrm{TiO}}_{2}$ films: (**a**) non-annealed, (**b**) PC-annealed, and (**c**) furnace-annealed; from [29].

Figure 2 shows AFM analysis of the surfaces of unannealed, furnace-annealed (FA), and PC-annealed ${\mathrm{TiO}}_{2}$ films. To explain the impact of PC on the optical, electrical, and structural characteristics of ${\mathrm{TiO}}_{2}$ films, prior research indicated that roughness is more significant in furnace and PC-annealed films compared to as-cast ${\mathrm{TiO}}_{2}$ films and the absence of anatase in untreated films [29]. Figure 2 shows that the as-cast films exhibit a smooth surface, which is attributed to the presence of small grains. On the other hand, the treated films display higher roughness and larger grains, suggesting improved crystallinity and conductivity. This confirms the performance of both PC-annealed and furnace-annealed ${\mathrm{TiO}}_{2}$ films (15.1–15%), as shown in Table 2. Another study examined the performance of PSCs on rigid and flexible substrates using a combination of the photonic annealing of ${\mathrm{TiO}}_{2}$ and pulverization of perovskite ${\mathrm{CH}}_{3}{\mathrm{NH}}_{3}{\mathrm{PbI}}_{3-x}{\mathrm{Cl}}_{x}$. The results showed a performance of 11.5% for PSCs on rigid substrates and 8.1% for flexible PSCs (see Table 2). The latter demonstrated a retention of initial performance exceeding 60% after 1000 bending cycles, displaying the compatibility of PSCs with lightweight and inexpensive flexible substrates. This suggests that PSCs are a feasible technique for roll-to-roll manufacturing [17].

**Figure 3:**
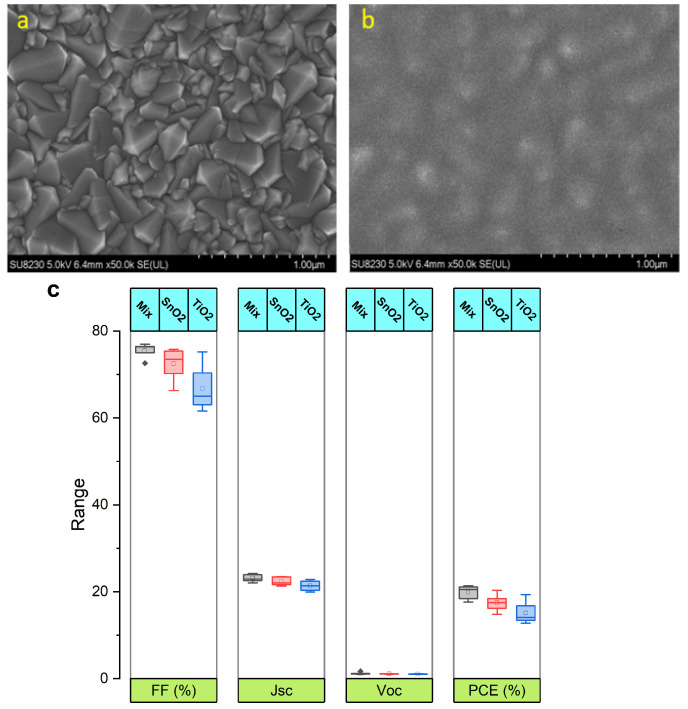
SEM image for (**a**) FTO and (**b**) SnO_2_ top surface after IPL treatment. (**c**) Box plot of the performance parameters of PSCs with different ETLs using the data from Table 3.

${\mathrm{SnO}}_{2}$ is a commonly used ETL in the fabrication of PSCs. It has received significant attention due to its remarkable stability under UV light, large bandgap, and greater electron mobility than ZnO and ${\mathrm{TiO}}_{2}$ [31,33]. Several approaches have proven the feasibility of fabricating planar PSCs based on solution treatment of ${\mathrm{SnO}}_{2}$ nanoparticles [34,35], atomic layer deposition (ALD) [36,37], and ${\mathrm{SnO}}_{2}$ sol-gel [38]. While these techniques have yielded high PCE, they necessitate extended annealing and growth periods, which could be a drawback for large-scale manufacturing. In this context, the IPL of ${\mathrm{SnO}}_{2}$ appears to be a promising and compatible technique that avoids high-temperature annealing and significantly reduces the annealing time. Preliminary research on ${\mathrm{SnO}}_{2}$’s PC has revealed a significant reduction in the roughness of ${\mathrm{SnO}}_{2}$ films before and after photonic annealing, from 116.6 nm to 82.8 nm [31], with a performance of 15.3% (Table 2). In a previous study, the IPL of the ${\mathrm{SnO}}_{2}$ films on the FTO substrates indicates that the grain boundaries previously present in the FTO film (Figure 3a) have disappeared, validating that the optimized IPL parameters should make the ${\mathrm{SnO}}_{2}$ film more compact and uniform (Figure 3b). Reducing the number of grain boundaries can increase shunt resistance and decrease recombination effects [32].

Table 3 displays PSCLs produced with identical techniques using different ETLs, ${\mathrm{TiO}}_{2}$, ${\mathrm{SnO}}_{2}$, or a combination of both (Mix). Based on the performance parameters of the PSCs reported in Figure 3c, we observe that ${\mathrm{SnO}}_{2}$ exhibits significantly better performance than ${\mathrm{TiO}}_{2}$, especially in terms of FF and Jsc. This is because ${\mathrm{SnO}}_{2}$ has advantageous properties such as a wide bandgap, high transparency, high mobility, and excellent stability [39,40], which are ideal for use as an ETL for planar PSCs. However, a ${\mathrm{TiO}}_{2}$ and ${\mathrm{SnO}}_{2}$ bilayer, or a mixture of ${\mathrm{SnO}}_{2}$ with ${\mathrm{TiO}}_{2}$ dopants, offers even better performance than ${\mathrm{SnO}}_{2}$. This is attributed to the excellent charge selectivity and low charge recombination in the interface perovskite/${\mathrm{SnO}}_{2}$ devices [36].

**Table 3:** Performance parameters of PSCs with ${\mathrm{TiO}}_{2}$, ${\mathrm{SnO}}_{2}$ or a mix of both.

ETL	Jsc (mA.cm^−2^)	$V_{\mathrm{oc}}$(V)	FF (%)	PCE (%)	Ref
${\mathrm{TiO}}_{2}$/${\mathrm{SnO}}_{2}$	20.8/21.3	0.99/1.05	61.6/66.3	12.8/14.8	[41]
ETL ^1^	19.92/21.63/22.06	1.09/1.11/1.02	65.54/70.24/72.6	14.18/16.92/18.46	[42]
ETL ^1^	22/22.1/22.9	0.99/1.14/1.2	64.5/75.4/76.4	14/19/21.1	[43]
ETL ^1^	22.85/23.45/23.91	1.13/1.14/1.69	75.2/75.8/76.5	19.33/20.34/21.4	[44]
${\mathrm{SnO}}_{2}$/Mix	23.4/24.2	1.03/1.1	75/77	18.09/20.5	[45]
${\mathrm{TiO}}_{2}$/Mix	22.06/22.58	1.01/1.04	72.4/75	16.16/17.64	[46]

^1^ ${\mathrm{TiO}}_{2}$/${\mathrm{SnO}}_{2}$/Mix.

**Figure 4:**
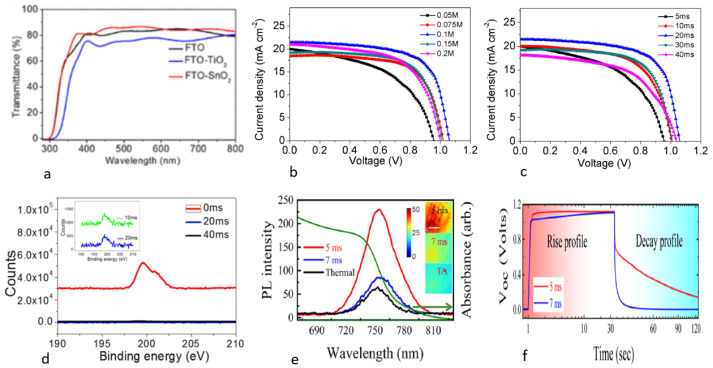
(**a**) Transmittance of FTO, ${\mathrm{TiO}}_{2}$, and ${\mathrm{SnO}}_{2}$ films [34]. The J-V curves for PSCs with ${\mathrm{SnO}}_{2}$ as ETL by (**b**) barying the concentration and (**c**) varying the PC duration. (**d**) XPS spectrum of the Cl 2p peak; the inset shows an enlarged view of the peak Cl 2p intensity [31]. (**e**) Evolution of PL intensity for devices utilizing the TA of ${\mathrm{SnO}}_{2}$ ETL film and PC with varying pulse duration. (**f**) Open circuit voltage decay (OCVD) for ${\mathrm{SnO}}_{2}$ PC-annealed devices at 5 ms and 7 ms [12].

Figure 4a displays the transmittance spectra of the FTO, FTO/${\mathrm{TiO}}_{2}$, and FTO/${\mathrm{SnO}}_{2}$ samples. It is evident that the ${\mathrm{SnO}}_{2}$ film has higher transmittance compared to the ${\mathrm{TiO}}_{2}$ film owing to its wider bandgap. This will enhance the Jsc current density and consequently the PCE. The optimization of photonic annealing time and ${\mathrm{SnO}}_{2}$ concentration are critical factors in improving ${\mathrm{SnO}}_{2}$ ETL crystallization. J-V curves for varying ${\mathrm{SnO}}_{2}$ concentrations and photonic annealing times can be seen in Figure 4b,c. To achieve optimal results, optimizing concentration and time sequencing is recommended. Figure 4d shows the XPS analysis of ${\mathrm{SnO}}_{2}$ films synthesized from the original ${\mathrm{SnCl}}_{4}$ solution. The figure shows a progressive decrease in the Cl 2p peak with increasing pulse duration, which finally disappears completely after a duration of 20 ms. This confirms that the chlorine is entirely photolyzed and ${\mathrm{SnCl}}_{4}$ is fully converted to ${\mathrm{SnO}}_{2}$ [31,47]. Several comparative studies of the electron collection efficiency of compact ${\mathrm{SnO}}_{2}$ and ${\mathrm{TiO}}_{2}$ layers using photoluminescence (PL) have confirmed that the PL quenching resulting from charge carrier extraction across the ETL/perovskite interface is significant for the ${\mathrm{SnO}}_{2}$ layer [48]. Figure 4e displays the photoluminescence analysis of ${\mathrm{SnO}}_{2}$ films’ TA and PC at various pulse durations. It can be observed that the PL quantum yield is significantly reduced for the 20 ms pulse duration compared to 5 ms. By optimizing energy density and pulse duration, quenching can be similar to or better than thermal annealing. The decrease in PL suggests more efficient separation of charge carriers within the perovskite layer and faster extraction and transfer facilitated by the compact ${\mathrm{SnO}}_{2}$ layers using PC annealing [40]. The high electron mobility and lower conduction band of ${\mathrm{SnO}}_{2}$ compact layers lead to efficient carrier dissociation in ${\mathrm{SnO}}_{2}$ PSCs [49,50].

Figure 4f shows the OCVD measurement for photonically annealed devices, which helps to understand the relevant changes in interface properties such as the recombination effect and trapping dynamics. We can see, in the light-off state, the high carrier concentration at the oxide–perovskite interface leads to rapid recombination [12], resulting in a faster drop in $V_{\mathrm{oc}}$, which can be observed for 7 ms devices. Improving pulse time and selecting an appropriate ETL can enhance PSC performance. The ${\mathrm{SnO}}_{2}$ plays an important role in charge separation (electron, hole) in the ${\mathrm{SnO}}_{2}$/perovskite interface. The accumulation of negative charges within the perovskite leads to a dielectric polarization at the interface, which has been able to maintain the $V_{\mathrm{oc}}$ values and allows a slow decay of the $V_{\mathrm{oc}}$ [51] when the light is turned off for devices fabricated with 5 ms pulses.

## 4. Photonic Curing of the Perovskite Layer

**Figure 5:**
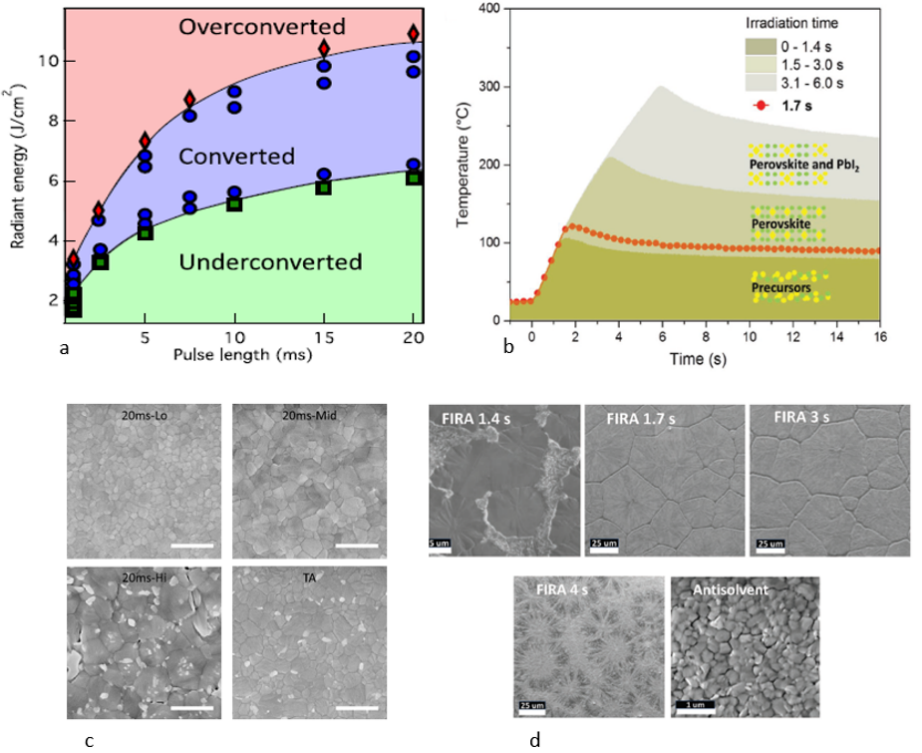
The three zones of perovskite crystallization can be defined through two methods: (**a**) PC, where the pulse duration is fixed and the energy density is varied from [10]; (**b**) Flash Infrared Annealing (FIRA), where the energy density is fixed while the pulse duration is varied [55]. SEM images for thermal and photonic annealing, respectively, for (**c**) aPC with increased energy density from [10]; (**d**) FIRA with increased pulse duration from [55].

Photonic curing has the potential to benefit from the exceptional optical properties and substantial light absorption capacity of perovskite materials. This could lead to a more compatible approach with IPL processes, which present a multitude of alternatives to traditional thermal methods. The benefit of utilizing the PC method for perovskite is that it can quickly sinter vast areas at high temperatures in a short period without altering the substrate’s composition, and it can be completely processed under ambient conditions. Studies indicate that the efficiency of PSC increases as the annealing temperature increases until 150 °C. Beyond this temperature, perovskite decomposes into ${\mathrm{PbI}}_{2}$ [52]. The IPL can reach temperatures high enough to induce a phase transition, but no degradation occurred, because the rate of thermal response was too rapid [53]. The improved quality of perovskite films depends on precise control of energy density and pulse duration to eliminate morphological defects, such as cracks or pinholes, in order to minimize series resistance and the possibility of charge recombination in PSC [53,54]. This review will examine how energy density and pulse duration affect crystallization, microstructure, and topography and thereby influence PSC performance. The optimal approach for perovskite crystallization using pulsed light involves setting one parameter, either pulse duration or energy, and adjusting the other to determine the zones where the films are partially converted, fully converted, or degraded, based on SEM, XRD or absorbance characterization. This leads to two graphs, temperature vs. time or energy density vs. pulse duration, as displayed in Figure 5a,b [10,55]. In both methods, increasing the energy density for PC or the pulse duration for FIRA leads to a rise in the annealing temperature. This phenomenon can produce partially converted perovskites due to the presence of the solvent, fully converted perovskites when the annealing temperature is optimized with the boiling point of the solvent, or overconverted perovskites due to MAI evaporation at high temperatures, resulting in the thermal degradation of the perovskite. Figure 5c,d depict SEM images of perovskite films treated with both thermal and photonic annealing processes. The results show that for both PC and FIRA, an increase in energy density or pulse duration leads to an increase in grain size. In the case of FIRA 1.3s Figure 5c, individual domains are separated by regions that are likely non-crystalline. However, pulse durations of 1.7 and 3 s cause increases in grain size, leading to large crystalline domains with no pinhole in the perovskite films. This can be attributed to the much slower nucleation. Increasing either the energy density (20 ms HI sample, Figure 5c) or the impulse time (FIRA 4s sample, Figure 5d) results in film degradation, causing structural defects like cracks and voids. The energy induced in the film must be precisely controlled to enable the complete crystallization of the film without causing perovskite degradation [55]. At the optimum energy densities and pulse durations for PC and FIRA, respectively, the film quality is comparable to or better than that of TA control films. The growth temperature’s control has a structural impact on the final crystal [56]. The pulse length and energy density are critical factors in controlling the nucleation density, which is imperative for enhancing the performance of PSCs.

**Figure 6:**
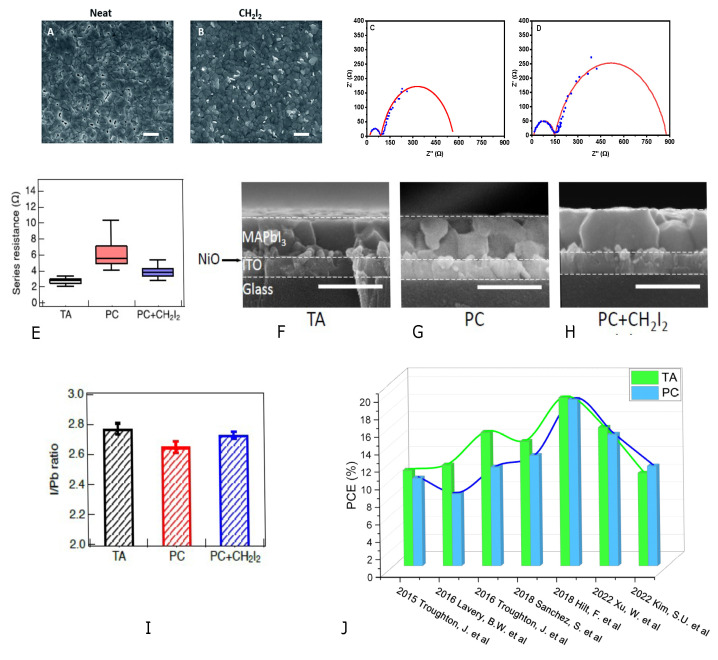
SEM images of photonic annealing of perovskite films: (**A**) neat, (**B**)+ additive ${\mathrm{CH}}_{2}I_{2}$, (**C**,**D**) Nyquist plots for films without and with ${\mathrm{CH}}_{2}I_{2}$ [57]. (**E**) Series resistance of devices annealed using a thermal annealing, PC, and PC with ${\mathrm{CH}}_{2}I_{2}$ additive and (**F**–**H**) SEM cross-section images of devices annealed using TA, PC, or PC with ${\mathrm{CH}}_{2}I_{2}$ additive [61]. (**I**) Comparison of I and Pb ratios in TA, PC, and PC+${\mathrm{CH}}_{2}I_{2}$ films utilizing the EDS technique from [61]. (**J**) PCE comparison for PC and TA since 2015 from ([52,55,61,62,63,64,65]) and data from Table 4.

Producing high-quality perovskite films in ambient conditions requires a precise precursor chemistry treatment. Binders and surfactants can improve film topography, but they can also leave organic residues that impair PSC performance. UV light, which is part of the IPL spectrum, can decompose these residues. In this section, we present the effect of PC on perovskite with an additive on the performance of PSCs. The performance of perovskite solar cells (PSCs) is significantly enhanced by its improved morphology. Various studies have suggested utilizing additives, including polymers, organic precursors, and alkyl halides, to enhance the morphology of perovskite films [57]. Adding diiodomethane (${\mathrm{CH}}_{2}I_{2}$) or diidooctane to the perovskite formulation enhances device efficiency. ${\mathrm{CH}}_{2}I_{2}$ decomposes into I^−^ and CH_2_I^+^ when irradiated with ultraviolet (UV) light [53]. Including iodine in the photonic annealing of perovskite should improve film quality by improving surface coverage, filling iodine vacancies in the crystal lattice, reducing roughness, and promoting uniform crystallites with growth orientations aligned compared to the film without, ${\mathrm{CH}}_{2}I_{2}$ [53,58], thus improving device performance. SEM images (Figure 6A,B,F–H) reveal a surface that is filled without any holes and with larger grains for the ${\mathrm{CH}}_{2}I_{2}$-treated films. The Nyquist diagram provides an overview of the film’s electrical properties, including changes in impedance or conductivity due to PC and the presence of ${\mathrm{CH}}_{2}I_{2}$. Figure 6C,D demonstrate that at high frequencies, the series resistance, which can be deduced from the shift of the first semicircle, is smaller for the film with ${\mathrm{CH}}_{2}I_{2}$ [59]. Moreover, in the low-frequency region, the second semicircle is wider, suggesting a higher recombination resistance [57]. This indicates slower charge carrier recombination, better charge separation, and reduced losses [60]. This implies that the addition of diiodomethane has improved the quality and reduced the defect density of the perovskite film.

**Table 4:** Photovoltaic parameters for perovskite devices fabricated by PC.

Year	Jsc (mA.cm^−2^)	$V_{\mathrm{oc}}$(V)	FF (%)	PCE (%)	Ref
2015	15.36	1.01	64.3	10	[62]
2016	16.55	1.02	69	11.5	[52]
2016	16	1.06	67	11.27	[63]
2018	22.7	1.11	74	19	[55]
2018	20.7	0.97	62	12.6	[64]
2022	19.37	1.05	73.4	15.04	[61]
2022	18.44	0.94	65.9	11.34	[65]

The EDX technique (Figure 6I) confirms a deficiency of iodine ions in the PC of the perovskite films compared to those annealed by TA and PC+${\mathrm{CH}}_{2}I_{2}$. However, the PC promotes photolysis, decomposing ${\mathrm{CH}}_{2}I_{2}$, which fills the holes and improves the crystallinity of the perovskite films. The addition of ${\mathrm{CH}}_{2}I_{2}$ promoted vertical grain growth, reduced series resistance, and improved PCE from 11.86% to 15.04%, which is similar to the TA device of 15.81% [61]. This study supports previous findings indicating that the use of alkyl-halide additives during the photonic curing process improves interactions between the perovskite precursor and solvent, leading to better surface coverage and higher PCE [32]. Figure 6J illustrates the evolution of PC perovskite device performance compared to TA over almost a decade of development progress. The data reveal a substantial improvement in the performance of PC devices compared to those utilizing TA. The reduction in PCE gap between TA and PC can be attributed to the enhanced mastery of this technique and a better understanding of the crystallization dynamics induced by PC in recent years. Furthermore, PC technology offers significant opportunities and many advantages over TA. The most notable advantages are the shorter processing time and compatibility with low-heat-tolerant [9], transparent substrates, as shown in Table 5. This suggests that PC annealing will outperform traditional techniques in the near future.

PC significantly impacts the conversion of perovskite from the precursor to the crystalline phase. PC affects the optical, crystalline, and morphological properties of the films [10]. Notably, PC reduces surface bulk defects and enhances light absorption, resulting in denser films and influencing the performance of PSCs [10,70]. Other studies also highlight PC’s role in defect mitigation and absorption enhancement [71]. Bulk defect attenuation improves the crystallinity and stability, as well as the alignment of energy levels, enabling efficient charge extraction [72]. Furthermore, a recent study has shown that the incorporation of one- and two-dimensional halide perovskites as novel passivants enhances the performance of perovskite solar cells [73]. The integration of these perovskites into the serigraphy process [74], combined with photonic annealing, has great potential for large-scale industrial applications.

The progress of perovskite-based solar cells has been significant, in part due to advancements in photonic processing [75]. In addition to work on transport layers and electronic contacts, techniques such as encapsulation [76], efficient surface nanostructuring [77], and optimized light management have improved both the efficiency and stability of perovskite-based cells [78]. These advances pave the way for more efficient, durable, and economically viable perovskite devices.

## 5. Conclusions

This paper presents a literature review on the rapid annealing of HTL (NiOx), ETL (${\mathrm{SnO}}_{2}$ and ${\mathrm{TiO}}_{2}$), and photoactive perovskite layers under ambient conditions. The photonic annealing of perovskite and ETL/FTL layers, which require high temperatures, enabled rapid sintering in the order of milliseconds with photovoltaic performance comparable to standard techniques. The addition of ${\mathrm{CH}}_{2}I_{2}$ has been shown to enhance the formation of perovskite morphology with larger grains by releasing iodine during the PC processes. This approach provides significant advantages for quick, cost-effective, R2R production. The integration of photonic curing techniques into PSCs has led to a notable enhancement in efficiency and performance, as evidenced by the refinement of morphology, crystallinity, and interface quality. However, challenges remain, including the complexity of experimentation for optimal curing and the limitations of material compatibility. The precise control of parameters such as energy density and duration is crucial to prevent the damage of layers. Further investigations are necessary to comprehend and explore the science and dynamics of thin film crystallization, as well as to extend the PC process to diverse materials for various perovskite solar cell (PSC) configurations and other applications. In summary, the evolution of PC has considerably enhanced the understanding of curing parameters, expanded its applicability to different substrates, improved control of layer morphology, and integrated it into manufacturing processes more effectively. These advances position PC as a fascinating tool in the process of perovskite solar cell manufacturing, offering substantial benefits for device efficiency and stability.

## Figures and Tables

**Table 1:** Comparison of the 3 configurations.

Annealing Process	NiOx	MAPI	PCEmax (%)
TA/TA	TA	TA	11.5
TA/PC	TA	PC	12.5
PC/PC	PC	PC	11.7

**Table 2:** Performance parameters of PSCs with ${\mathrm{TiO}}_{2}$, ${\mathrm{SnO}}_{2}$ or a mix of both.

Annealing Process	Energy Density Tension Energy	Pulse Length (ms)	$V_{\mathrm{oc}}$ (V)	$J_{\mathrm{sc}}$ (mA cm^−2^)	FF (%)	PCE (%)	Configuration	Ref
Glass-PC	12.3 J.cm^−2^	2	1.05	20.2	78.5	16.7	ITO/compact${\mathrm{TiO}}_{2}$/MAPI Spiro-OMetad/ Au	[28]
Glass-TA	-	-	1.05	20.2	76.5	16.3		
PEN-PC	2.35 J.cm^−2^	2	1.05	18.2	64.1	12.3		
Glass-PC	200V	7	1.04	19.9	60.9	15	ITO/${\mathrm{TiO}}_{2}$/${\mathrm{CH}}_{3}{\mathrm{NH}}_{3}{\mathrm{PbI}}_{3-x}{\mathrm{Cl}}_{x}$/ Spiro-OMetad/Au	[29]
Glass-FA	-	-	1.04	19.4	65.7	15.1		
PET-PC	200V	7	1.09	16.9	61	11.2		
Glass-TA	-	2	1	18	61.2	11.5	ITO/${\mathrm{TiO}}_{2}$/${\mathrm{CH}}_{3}{\mathrm{NH}}_{3}{\mathrm{PbI}}_{3-x}{\mathrm{Cl}}_{x}$/ Spiro-OMetad/Ag	[17]
PET-PC	17.3 J.cm^−2^	2	1.03	15.3	51.4	8.1		
Glass-PC	46 J.cm^−2^	20	1.06	21.4	67	15.3	FTO/${\mathrm{SnO}}_{2}$/MAPI/PTAA/Au	[31]
Glass-PC	2100 J	2	1.02	15.78	78.3	12.56	FTO/${\mathrm{SnO}}_{2}$/MAPI/PTAA/Au	[32]
PET-PC	2100 J	2	0.99	11.33	64.75	7.6	ITO/${\mathrm{SnO}}_{2}$/MAPI/PTAA/Au	
Glass-PC	11.3 J.cm^−2^	7	1.14	22.7	80.4	21.1	FTO/${\mathrm{SnO}}_{2}$/(${\mathrm{FA}}_{0.83}{\mathrm{MA}}_{0.17}{)}_{0.95}$ ${\mathrm{Cs}}_{0.05}{\mathrm{PbI}}_{2.5}{\mathrm{Br}}_{0.5}$/ Spiro-OMetad/Au	[12]
Glass-TA	-	-	1.12	22.5	79.8	20.2		

**Table 5:** Advantages and disadvantages of PC and TA.

Aspect	PC	TA	Ref
**Advantages**			
Speed processing	Rapid (ms-s)	Longer (min-h)	[66]
Heat exposure reduction	Significantly reduces	High temperature requierd	[7]
Heat sensitive substrat	Minimal risk	Potentiel risk	[2]
Temperature control	More presice	Must be carfully regulated to avoid damage	[67]
Selective processing	Yes	No	[68]
Equilibrated heating	No	Yes	[10]
**Disadvantages**			
Initial cost	Higher due to specialized equipment	Lower	[69]
Process complexity	May require additional technical expertise	Process may be simpler	

## Data Availability

Data are contained within the article.

## Abbreviations

The following abbreviations are used in this manuscript:

PSCs	Perovskite solar cells
PC	Photonic curing
TA	Thermal annealing
FA	Furnace annealing
ETL	Electron transport layer
HTL	Hole transport layer
